# Incongruence Between Prerequisite Molecular Testing and Treatment with Personalized Therapies for Non-Small Cell Lung Cancer: A Surveillance, Epidemiology and End Results-Medicare Study

**DOI:** 10.3390/ijms26104581

**Published:** 2025-05-10

**Authors:** Wiley M. Turner, Stephanie Tuminello, Matthew Untalan, Raja Flores, Emanuela Taioli

**Affiliations:** 1 Institute for Translational Epidemiology, Icahn School of Medicine at Mount Sinai, New York, NY 10029, USA; wiley.turner@mountsinai.org (W.M.T.); stephanie.tuminello@mountsinai.org (S.T.); matthew.untalan@mountsinai.org (M.U.); 2 Department of Thoracic Surgery, Icahn School of Medicine at Mount Sinai, New York, NY 10029, USA; raja.flores@mountsinai.org; 3 Tisch Cancer Institute, Icahn School of Medicine at Mount Sinai, New York, NY 10029, USA

**Keywords:** non-small cell lung cancer (NSCLC), personalized medicine, molecular diagnostic testing, immunotherapy, targeted therapy

## Abstract

Personalized medicine, including targeted and immunotherapy, has substantially changed the landscape of non-small cell lung cancer (NSCLC). About 30% of NSCLC tumors have targetable mutations, like EGFR, and 28% of tumors will have a PD-L1 score or tumor mutation burden high enough to warrant immunotherapy; a molecular test prior to therapy assesses eligibility. However, the congruence between testing and treatment, and the effect of treatment without testing on outcomes, is understudied. We extracted a cohort of NSCLC patients from the Surveillance, Epidemiology and End Results (SEER)-Medicare-linked data. The primary outcome was survival. Demographic and clinical characteristics of those receiving vs. not receiving a molecular test were compared, and a Kaplan–Meier curve along with a multivariable Cox proportional hazards regression assessed the association of survival with the receipt of molecular diagnostic testing, adjusting for sex, race, age, income tract, metro/urban/rural region, histology, and stage. There were 911 NSCLC patients treated with personalized therapy, of which 513 (56.3%) had received prior molecular testing. Black patients were less likely than White patients to receive testing (36.4% vs. 59.9%; *p* < 0.001). Testing was significantly more frequent in patients with fewer comorbidities (*p* < 0.001), adenocarcinoma (*p* = 0.004), tumors in the lower lobe (*p* = 0.037), or diagnosed at a later stage (*p* = 0.026). Only 39.9% of patients receiving EGFR inhibitors were initially tested for an EGFR mutation. Among NSCLC patients treated with personalized therapy, untested patients were at increased mortality risk compared to tested patients (median survival 8.22 vs. 12.79 months, *p* = 0.024). After adjustment, treated patients not tested beforehand had a significantly higher mortality risk (HRadj: 1.20; 95% CI: 1.04–1.40). Substantial incongruence between receipt of molecular testing and personalized therapies exists according to nationwide claims data, with only about half of treated patients having had an appropriate molecular diagnostic workup. This could explain, in part, the higher mortality among treated patients who did not undergo testing. Before expanding personalized therapies, further addressing these issues through standardized, reflexive testing protocols and improved clinician education is critical to optimizing the integration of molecular diagnostics into treatment planning.

## 1. Introduction

Lung cancer is one of the most prevalent and deadly cancers in the US, with most diagnoses occurring only after the cancer has metastasized, yet stage IV patients only have a survival rate of 8.2% [1]. In the past, non-small cell lung cancer (NSCLC) patients, the most common histologic group accounting for about 80% to 85% of all lung cancer cases, have traditionally been treated with chemotherapy and radiation therapy in some combinations [2]. However, over recent years, the rise in targeted and immunotherapies has significantly altered the outcomes of late-stage NSCLC patients, allowing for a significant improvement in their survival [3]. These types of personalized medicine treatments rely on molecular biomarkers to assess eligibility. Approximately 30% of all NSCLC patients, including 60% of adenocarcinomas and 50–80% of squamous cell carcinomas, have targetable mutations in receptors or protein kinases that drive tumor proliferation [4,5,6]. The most common target is the epidermal growth factor receptor (EGFR) tyrosine kinase pathway, which is treated with tyrosine kinase inhibitors (TKIs) [6]. The anaplastic lymphoma kinase (ALK), BRAF, KRAS, and ROS1 pathways are other oncogenic pathways that can be affected by targeted therapies [7]. Immunotherapies mostly work on the programmed death 1-programmed death ligand 1 (PD-1-PD-L1) and cytotoxic T-lymphocyte-associated antigen 4 (CTLA-4) pathways [8]. Positive PD-L1 expression on the tumor surface serves as an indicator that a patient may be responsive to PD-1-PD-L1-targeting drugs, with CTLA-4 presence on T cells serving as a marker that a patient may be responsive to anti-CTLA-4 immunotherapeutic drugs [8,9]. Additionally, in scenarios when PD-L1 and CTLA-4 expression is low, high tumor mutational burden (TMB) can also serve as a biomarker for immunotherapy response, with a higher TMB resulting in more neo-antigens that enhance T-cell reactivity and improve treatment outcomes [10,11]. There are molecular testing methods to assess if a patient is a candidate for targeted therapies, performed on tumor tissue or blood samples through techniques such as PCR, immunohistochemistry (IHC), or next-generation sequencing (NGS)-based gene panels [12,13]. These methods are generally recommended for patients with advanced or metastatic NSCLC, with the results informing the patient’s treatment eligibility based on the biomarkers identified [14].

While personalized medicine has yielded promising advancements in NSCLC treatment, there has recently been a push towards using these “personalized” treatments more broadly, even without the prerequisite molecular diagnostic testing. For instance, the FDA has recently approved the use of immunotherapy in conjunction with chemotherapy across all strata of tumor PD-L1 expression levels, after overall survival (OS) was shown to improve with treatment, even among those with <1% tumor PD-L1 expression [15,16,17]. Yet, congruence between appropriate molecular diagnostic workup and subsequent personalized medicine treatment is understudied; moreover, the efficacy of these treatments in untested and potentially ineligible patients warrants additional investigation using real-world, patient-level data in addition to clinical trials. The primary objectives of this study were to investigate the rate of molecular diagnostic tests among treated NSCLC patients and to investigate whether, among patients who have received targeted or immunotherapy, those who were not diagnostically tested prior to treatment derived the same benefit towards survival from the personalized therapies as those who were tested.

## 2. Results

There were 911 stage III and IV NSCLC patients who were treated with targeted or immunotherapy, of which 513 (56.3%) received some type of molecular diagnostic testing. Compared to treated White patients, fewer treated Black patients received a molecular diagnostic test before treatment (36.4% vs. 59.9%; *p* < 0.001). Patients who had fewer comorbidities (*p* < 0.001), adenocarcinoma (*p* < 0.001), tumors in the lower lobe (*p* = 0.037) or were diagnosed at a later stage (*p* = 0.026) were more likely to have molecular testing before treatment (Table 1).

Among the 513 patients who received some kind of molecular testing as well as a targeted or immunotherapy treatment, 416 (81.1%) received EGFR treatment. Among these patients, only 166 (39.9%) received the corresponding precursor EGFR molecular test (Table 2).

### Association of Molecular Testing with Survival

**Figure 1:**
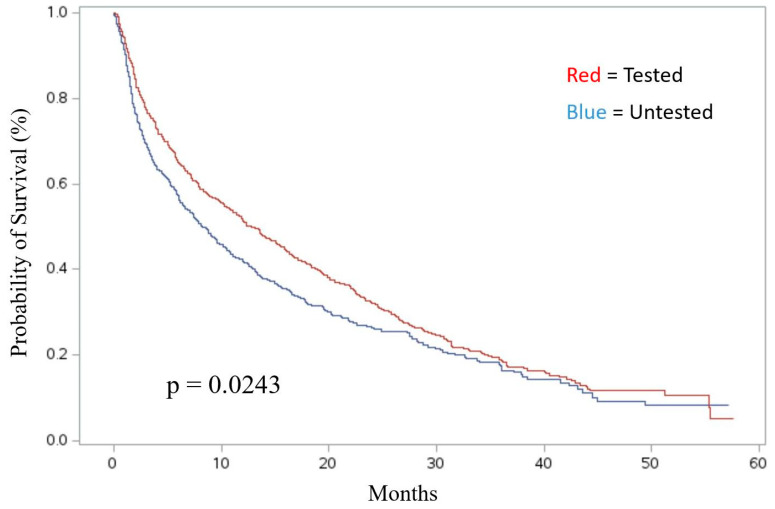
Survival of targeted- and immunotherapy-treated NSCLC patients, stratified by receipt of molecular testing (measured from first date of treatment).

Receiving molecular diagnostic testing was positively associated with OS among treated patients. In the univariate analysis, patients who underwent molecular diagnostic testing had a median survival time of 12.79 months compared to patients who were untested, who had a median survival time of 8.22 months (*p* = 0.0243) (Figure 1). This difference held true in the adjusted model, where treated patients who did not receive genetic testing had a statistically significant higher risk of death (HR_adj_ [hazards ratio]: 1.20; 95% CI: 1.04–1.40) (Supplementary Table S3).

## 3. Discussion

A disproportionate underutilization of molecular diagnostic testing across racial and socioeconomic strata exists, with a lack of diagnostic testing being associated with worse patient outcomes among advanced NSCLC patients [12,18,19,20]. This testing serves as the cornerstone of personalized oncology, enabling clinicians to identify actionable gene or protein expressions that can guide treatment. This level of precision not only increases the likelihood of treatment efficacy by making sure that the treatment marker is, in fact, present, but also minimizes ineffective therapies and unnecessary toxicity. While addressing the disparity in testing is important, it is also crucial to examine if there is congruence between diagnostic testing and its associated treatment, and how treatment efficacy may differ among tested vs. untested patients who undergo personalized medicine treatments. Here, we show that among NSCLC patients treated with targeted therapies or immunotherapy, there is a significant incongruence in receiving a molecular diagnostic test and receiving its associated treatment, as almost half of all treated patients did not receive any prerequisite testing to assess eligibility, with upwards of 60% of patients treated with EGFR inhibitors, specifically, being untested for the EGFR mutation prior to treatment. This is especially concerning because patients who were tested before receiving personalized therapy had significantly better outcomes.

These findings align with previous studies highlighting a gap in the precise application of personalized medicine, as well as the general treatment of NSCLC patients with targeted or immunotherapies without the prerequisite confirmation of actionable mutations or expressions. For instance, data examining EGFR-tested patients show that, while about 33.6% of patients with the targetable EGFR mutation received erlotinib, a TKI, it was also given to 5.9% of patients without the EGFR mutation, affirming that these “targeted” approaches are not always as precise as originally intended [21,22]. In single- or multiple-institution studies, the proportion of advanced NSCLC patients with molecular testing results available ranged from 21% to 41%, with only about 8% having fully followed established testing recommendations; often, there was no documented reason for lack of testing [23,24]. The gap could stem from various challenges related to molecular diagnostic testing. For example, large disparities exist in healthcare infrastructure, especially in community practices with limited access, providing an initial barrier for many to access the testing [25]. Clinicians may also feel pressured to initiate the targeted treatment quickly to prevent any delays caused by waiting for certain molecular test results or due to limited access to comprehensive genomic profiling [26]. However, due to the precise mechanistic biological nature of these personalized treatments, which require patients to exhibit a specific molecular presence or deficiency to be effective, it is unsurprising that patients who do not undergo the necessary diagnostic testing end up having worse outcomes than those confirmed to have the biomarker [5,8,27]. Additionally, improper prescription of these treatments may delay the initiation of more effective, appropriate treatments, potentially wasting valuable time in managing the cancer and ultimately compromising the outcome [28]. Targeted therapies and immunotherapies can also have unwanted toxic effects on patients. For example, even in patients who do not exhibit PD-L1 or CTLA-4 biomarkers, immunotherapy drugs can have a wide variety of side effects, ranging from mild rashes to more severe CNS damage, and, in some cases, even death [29,30]. Guidance from the National Comprehensive Cancer Network (NCCN), which removed TKI use for those without EGFR mutations from their guidelines in 2015, and the FDA, which restricted erlotinib use to those with only certain EGFR mutations in 2016, has generally moved towards a testing-first philosophy [31]. While steps have already been taken, further addressing these issues through standardized, reflexive testing protocols and improved clinician education is critical to optimizing the integration of molecular diagnostics into treatment planning.

This study has several strengths: because the SEER-Medicare dataset was derived from nation-wide, patient-level data, we were able to create a population-based cohort of diverse NSCLC patients. With the specificity involved with the Medicare claims data, we were able to define the general receipt of diagnostic tests and treatment and individually stratify by type of test or treatment to investigate pairings, such as EGFR, more clearly. Additionally, we were able to adjust for important confounding factors such as comorbidities, race, or income tract, and directly examine the correlation of molecular diagnostic testing with patient survival—a challenging task when using clinical or electric health record data. However, while the SEER-Medicare dataset provides many strengths, it also has limitations. We could not stratify patients by treatment eligibility, as SEER-Medicare does not report diagnostic testing results. Future research should incorporate this information to better assess the differences in treatment efficacy between tested and untested patients. In addition, although mutation testing is recommended by the College of American Pathologists (CAP), the International Association for the Study of Lung Cancer (IASLC), and the Association for Molecular Pathology (AMP) for all patients with advanced NSCLC, some patients in our cohort may have had contraindications, insufficient tissue, or received testing not billed through Medicare—all scenarios not captured in SEER-Medicare [14]. Also, although we adjusted for many key confounders, SEER-Medicare does not report factors such as smoking status, occupational exposure, or provider-level differences; the impacts of demographic and clinical factors not captured in this dataset on testing incongruence need to be explored using other data sources. Tumor aggressiveness may also serve as a confounder, as clinicians might skip diagnostics and proceed directly to treatment in the most extreme cases. Future work should explore hospital-level data that can capture these details. Finally, the number of patients treated with targeted or immunotherapy was small, limiting the ability to conduct stratified analyses beyond Black and White patients [32]. For instance, we were unable to look at the testing rates and associated survival for Asian patients who have a markedly higher rate of EGFR mutations than White patients, approximately 50% vs. 20%, respectively, in adenocarcinoma cases [33]. As targeted and immunotherapy have become more widespread in use and popularity over the past decade, with future larger SEER-Medicare datasets, research should seek to directly examine personalized testing and treatment among these less represented groups.

While there has been a large push within the scientific community to discover novel treatments and therapies, there is not enough effort being performed to ensure that existing treatments are working at maximum efficiency. We have shown here that a disconnect exists between the supposed diagnostic tests and their associated therapy, with nearly half of the treated patients undergoing no kind of diagnostic test at all, and with these untested patients having significantly worse outcomes. To close this gap, it is important to focus on building testing infrastructure so that it becomes accessible to patients across the country, even in resource-limited areas, and on improving clinician education alongside the installation of reflexive protocols, upon which molecular testing can be initiated at diagnosis and the appropriate personalized treatment can then be prescribed. While this study contains data from 2013 to 2015, the work is still very much relevant, with groups like the Illinois Lung Cancer Collective working towards improving the quality of NSCLC testing through elevated guidelines and improved adherence [34]. Looking forward, it is important to continue to take these steps to understand and address the issue of testing incongruence to help close gaps in cancer care before focusing on en masse expansion of personalized treatments.

## 4. Materials and Methods

### 4.1. Study Design

**Figure 2:**
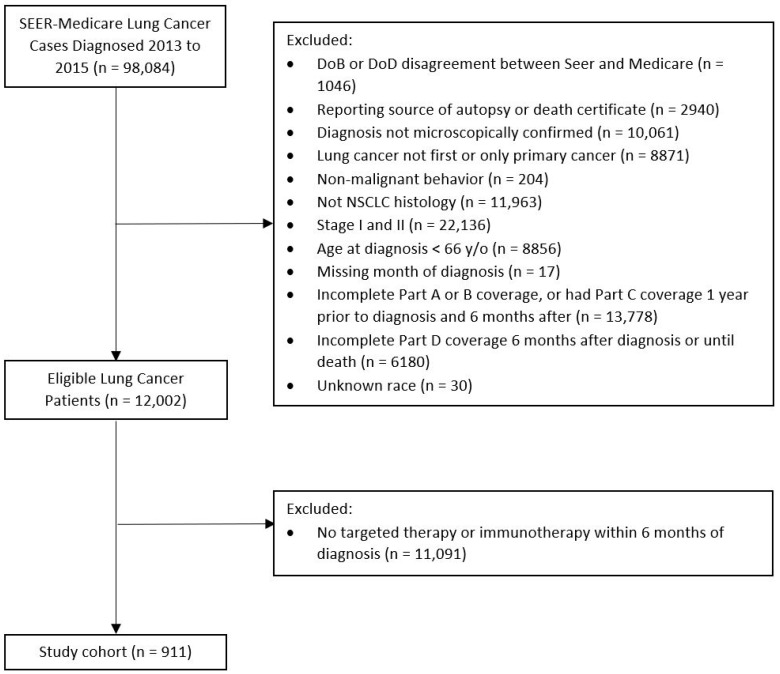
SEER-Medicare cohort selection. SEER, Surveillance, Epidemiology and End Results; DoB, date of birth; DoD, date of death; and NSCLC, non-small cell lung cancer.

A cohort of NSCLC patients was extracted from the Surveillance, Epidemiology and End Results (SEER)-Medicare-linked data. SEER includes cancer incidence and mortality data from population-based registries that cover approximately 35% of the United States population, and Medicare includes healthcare claims data for eligible beneficiaries, specifically those 65 years and older [35]. Patients included in this analysis must have had a microscopically first or only primary confirmed diagnosis of NSCLC diagnosed between 2013 and 2016, with 2013 marking the time of publication of evidence-based guidelines for the molecular analysis of lung cancers, which were established to guide treatment decisions with targeted inhibitors by the College of American Pathologists, the International Association for the Study of Lung Cancer, and the Association for Molecular Pathology to set standards [36]. The analysis was restricted to patients treated with targeted or immunotherapy. Patients were defined as having received targeted therapy if they had a Part D claim for any of the drugs listed in Supplementary Table S1 [36]. Patients were defined as having received immunotherapy if their claims records reflected the codes of C9483, C9453, J9299, J9027, C9284, or J9228 for PD-L1 or CTLA treatment. To properly capture comorbidities prior to diagnosis, patients were limited to those who were at least 66 years old at diagnosis, with continuous Part A and B coverage and no Part C coverage, for 1 year pre-diagnosis and 6 months post-diagnosis to capture all molecular testing events. Patients were also restricted to those with continuous Part D coverage 6 months post-diagnosis to capture the targeted and immunotherapy events (Figure 2). These restrictions also ensured that the patients were continuously covered by Medicare, so that missing data were not incorrectly coded as a lack of a comorbidity, diagnostic testing, or treatment. Patients were then further limited to those with stage III/IV as both targeted and immunotherapies are more frequently offered to those with advanced disease (Figure 2). The use of the SEER-Medicare dataset was approved by the Institutional Review Board of the Mount Sinai School of Medicine, in accordance with Mount Sinai’s Federal Wide Assurances (FWA#00005656, FWA#00005651) and a waiver of informed consent was obtained for all patients as part of the IRB application (STUDY-19-00500-CR003).

### 4.2. Main Predictors and Covariates

Data on patient characteristics, including age at diagnosis, sex, race, place of residence, histology, and marital status, were obtained from the SEER database. Sex was categorized as male or female. Race was classified into three categories: Black, White, and Other (representing all races aside from Black or White). Socioeconomic status, though not directly included in SEER, was determined using the Census Tract Poverty Indicator variable from the Patient Entitlement and Diagnosis Summary File (PEDSF). High-poverty areas are defined as census tracts in which 10% or more of the population lives below the poverty line. SEER also categorizes patients as living in either metro, urban, and rural regions based on the county’s population size at the time of diagnosis, following the Rural–Urban Continuum Codes, as defined by the United States Department of Agriculture (USDA) [37]. Marital status was classified as either married (including those in domestic partnerships) or unmarried (including those who have never married, been divorced, or are widows). Tumor histology was classified according to the International Agency for Research on Cancer (IARC) codes as squamous cell carcinoma, adenocarcinoma, or other [38]. The Charlson Comorbidity Index (CCI) scores were derived from claims data, following a modified version of the NCI CCI with ICD-9 and -10 codes [39]. Specifically, the CCI is defined by myocardial infarction, congestive heart failure, peripheral vascular disease, cerebrovascular accident or transient ischemic attack, chronic obstructive pulmonary disease, peptic ulcer disease, paralysis or hemiplegia, diabetes history, liver disease and kidney disease, with the latter three conditions further weighted by severity. Year of diagnosis was either 2013, 2014 or 2015. Patients were defined as having received a molecular test if their records reflected a claim occurring after the date of lung cancer diagnosis, corresponding to any of the Current Procedural Terminology Codes (CPT) reported in the Supplementary Table, and 83912 in the Outpatient claims file (Supplementary Table S2).

### 4.3. Primary Outcome

The primary outcome of interest was patient survival. Patients were tracked for this from their date of first treatment according to SEER-Medicare claims records. Patient survival time was then calculated using this initial treatment date alongside the patient’s recorded date of death, or, in cases where the patient lived until at least 31 December 2017—our last follow-up date—it was assumed that the patients were still living, and they were censored in the model.

### 4.4. Statistical Analysis

Descriptive statistics were performed for the socioeconomic, clinical and demographic variables. The associations between the explanatory and outcome variables were evaluated using Pearson’s chi-square tests for categorical variables and Student *t*-tests for continuous variables. Descriptive statistics with regard to EGFR treatment and testing were also performed among the broader tested cohort as a case study on the association between patients receiving a specific molecular test and then receiving the associated treatment.

A Kaplan–Meier analysis was conducted to initially establish a relationship between molecular testing and mortality among the entire cohort. An adjusted Cox proportional hazards regression model was then used to further evaluate the association of receipt of molecular diagnostic testing with overall survival (OS). The model was adjusted by race, census income tract, urban/rural region, sex, age, histology, stage, and Charlson comorbidity score, all confounders associated with NSCLC survival. The entire data analysis process was performed using SAS software version 9.4 [18].

## Figures and Tables

**Table 1:** Summary of all personalized medicine-treated patient characteristics according to receipt of molecular testing.

	Molecular Diagnostic Test		
	***No*** *n = 398 (43.7%)*	***Yes*** *n = 513 (56.3%)*	*p-value*
**Race**			<0.001
Black	28 (7.0%)	239 (3.1%)	
White	270 (67.8%)	403 (78.6%)	
Other	100 (25.1%)	94 (18.3%)	
**Census Tract Poverty Indicator (% in poverty)**			0.183
<10% in poverty	205 (51.5%)	287 (56.0%)	
>=10% in poverty	193 (48.5%)	226 (44.1%)	
**Residence ^b^**			0.018
Metro (>20k)	360 (91.1%)	450 (88.4%)	
Urban/Rural (<20k)	35 (8.9%)	59 (11.5%)	
**Sex**			0.252
Male	120 (30.2%)	173 (33.7%)	
Female	278 (69.9%)	340 (66.3%)	
**Age at diagnosis (years)**			0.800
65–69	72 (18.1%)	101 (19.7%)	
70–74	99 (24.9%)	127 (24.8%)	
75–79	85 (21.4%)	109 (21.3%)	
80–84	67 (16.8%)	94 (18.3%)	
85+	75 (18.8%)	82 (16.0%)	
**Marital Status**			0.393
Single	183 (46.0%)	213 (41.5%)	
Married	201 (50.5%)	282 (55.0%)	
Unknown	14 (3.5%)	18 (3.5%)	
**Charlson comorbidity score** (mean, sd ^a^)	1.3 (1.6)	1.0 (1.3)	<0.001
**Histology**			0.004
Squamous	35 (8.8%)	42 (8.2%)	
Adenocarcinoma	316 (79.4%)	444 (86.6%)	
Large cell/Other	47 (11.8%)	27 (5.2%)	
**Primary Tumor Sites**			0.037
Upper lobe	202 (50.8%)	246 (48.0%)	
Middle lobe	12 (3.0%)	21 (4.1%)	
Lower lobe	92 (23.1%)	155 (30.2%)	
Other	92 (23.1%)	91 (17.7%)	
**Stage**			0.026
III	109 (27.4%)	108 (21.1%)	
IV	289 (72.6%)	405 (78.9%)	
**Year of Diagnosis**			0.033
2013	128 (32.2%)	159 (31.0%)	
2014	134 (33.7%)	139 (27.1%)	
2015	136 (34.2%)	215 (41.9%)	

^a^ sd = Standard deviation; ^b^ residence missing for 7 patients.

**Table 2:** EGFR testing vs. EGFR treatment among tested patients.

	EGFR Test	
	***No*** *n = 316 (61.6%)*	***Yes*** *n = 197 (38.4%)*
**EGFR Treatment**		
No	66 (20.9%)	31 (15.7%)
Yes	250 (79.1%)	166 (84.3%)

## Data Availability

Researchers can apply to use the new data linkage using the SEER-Medicare data request process at https://healthcaredelivery.cancer.gov/seermedicare/obtain/requests.html (accessed on 1 March 2024).

## Supplementary Materials

The following supporting information can be downloaded at: https://www.mdpi.com/article/10.3390/ijms26104581/s1.
