# Supplementary material for: Incongruence Between Prerequisite Molecular Testing and Treatment with Personalized Therapies for Non-Small Cell Lung Cancer: A Surveillance, Epidemiology and End Results-Medicare Study
Source: Int J Mol Sci. 2025 May 10;26(10):4581. doi: 10.3390/ijms26104581 (PMC12111554; doi:10.3390/ijms26104581)
Supplement: Supplementary file 1 [file ijms-26-04581-s001.zip › ijms-3596573-supplementary.pdf]

**Table S1:** Targeted Therapy Drugs

| Site | Drug |
|---|---|
| EGFR | Erlotinib HCL |
| | Gefitinib |
| | Afatinib Dimaleate |
| | Osimertinib Mesylate |
| ALK | Alectinib HCL |
| | Crizotinib |
| | Ceritinib |
| ROS1 | Crizotinib |
| | Ceritinib |
| BRAF | Dabrafenib Mesylate |
| | Trametinib Dimethyl Sulfoxide |

**Table S2:** Billing Codes for Molecular Tests

| CPT Code | Description |
|---|---|
| 81235 | EGFR (epidermal growth factor receptor) (eg, non-small cell lung cancer) gene analysis, common variants (eg, exon 19 LREA deletion, L858R, T790M, G719A, G719S, L861Q) |
| 81275 | KRAS (Kirsten rat sarcoma viral oncogene homolog) (eg, carcinoma) gene analysis; variants in exon 2 (eg, codons 12 and 13) |
| 81276 | KRAS (Kirsten rat sarcoma viral oncogene homolog) (eg, carcinoma) gene analysis; additional variant(s) (eg, codon 61, codon 146) |
| 81400 | MOLECULAR PATHOLOGY PROCEDURE LEVEL 1 |
| 81401 | MOLECULAR PATHOLOGY PROCEDURE LEVEL 2 |
| 81402 | MOLECULAR PATHOLOGY PROCEDURE LEVEL 3 |
| 81403 | MOLECULAR PATHOLOGY PROCEDURE LEVEL 4 |
| 81404 | MOLECULAR PATHOLOGY PROCEDURE LEVEL 5 |
| 81405 | MOLECULAR PATHOLOGY PROCEDURE LEVEL 6 |
| 81406 | MOLECULAR PATHOLOGY PROCEDURE LEVEL 7 |
| 81407 | Molecular pathology procedure, Level 8 |
| 81408 | MOLECULAR PATHOLOGY PROCEDURE LEVEL 9 |
| 81415 | Exome (eg, unexplained constitutional or heritable disorder or syndrome); sequence analysis |
| 81416 | Exome (eg, unexplained constitutional or heritable disorder or syndrome); sequence analysis, each comparator exome (eg, parents, siblings) (List separately in addition to code for primary procedure) |
| 81417 | Exome (eg, unexplained constitutional or heritable disorder or syndrome); re-evaluation of previously obtained exome sequence (eg, updated knowledge or unrelated condition/syndrome) |
| 81425 | Genome (eg, unexplained constitutional or heritable disorder or syndrome); sequence analysis |
| 81426 | Genome (eg, unexplained constitutional or heritable disorder or syndrome); sequence analysis, each comparator genome (eg, parents, siblings) (List separately in addition to code for primary procedure) |
| 81427 | Genome (eg, unexplained constitutional or heritable disorder or syndrome); re-evaluation of previously obtained genome sequence (eg, updated knowledge or unrelated condition/syndrome) |

| | |
|---|---|
| 81445 | Targeted genomic sequence analysis panel, solid organ neoplasm, DNA analysis, and RNA analysis when performed, 5-50 genes (eg, ALK, BRAF, CDKN2A, EGFR, ERBB2, KIT, KRAS, NRAS, MET, PDGFRA, PDGFRB, PGR, PIK3CA, PTEN, RET), interrogation for sequence variants and copy number variants or rearrangements, if performed |
| 81455 | Targeted genomic sequence analysis panel, solid organ or hematolymphoid neoplasm, DNA analysis, and RNA analysis when performed, 51 or greater genes (eg, ALK, BRAF, CDKN2A, CEBPA, DNMT3A, EGFR, ERBB2, EZH2, FLT3, IDH1, IDH2, JAK2, KIT, KRAS, MLL, NPM1, NRAS, MET, NOTCH1, PDGFRA, PDGFRB, PGR, PIK3CA, PTEN, RET), interrogation for sequence variants and copy number variants or rearrangements, if performed |
| 81479 | Unlisted molecular pathology procedure |
| 81504 | Oncology (tissue of origin), microarray gene expression profiling of > 2000 genes, utilizing formalin-fixed paraffinembedded tissue, algorithm reported as tissue similarity scores |
| 81525 | Oncology (colon), mRNA, gene expression profiling by realtime RT-PCR of 12 genes (7 content and 5 housekeeping), utilizing formalin-fixed paraffin-embedded tissue, algorithm reported as a recurrence score |
| 81540 | Oncology (tumor of unknown origin), mRNA, gene expression profiling by real-time RT-PCR of 92 genes (87 content and 5 housekeeping) to classify tumor into main cancer type and subtype, utilizing formalin-fixed paraffinembedded tissue, algorithm reported as a probability of a predicted main cancer type and subtype |
| 81599 | Unlisted multianalyte assay with algorithmic analysis |
| 83890 | Molecular diagnostics; molecular isolation or extraction, each nucleic acid type (ie, DNA or RNA) |
| 83891 | Molecular diagnostics; isolation or extraction of highly purified nucleic acid, each nucleic acid type (ie, DNA or RNA) |
| 83892 | Molecular diagnostics; enzymatic digestion, each enzyme treatment |
| 83893 | Molecular diagnostics; dot/slot blot production, each nucleic acid preparation |
| 83894 | Molecular diagnostics; separation by gel electrophoresis (eg, agarose, polyacrylamide), each nucleic acid preparation |
| 83896 | Molecular diagnostics; nucleic acid probe, each |
| 83897 | Molecular diagnostics; nucleic acid transfer (eg, Southern, Northern), each nucleic acid preparation |
| 83898 | Molecular diagnostics; amplification, target, each nucleic acid sequence |
| 83900 | Molecular diagnostics; amplification, target, multiplex, first 2 nucleic acid sequences |
| 83901 | Molecular diagnostics; amplification, target, multiplex, each additional nucleic acid sequence beyond 2 (List separately in addition to code for primary procedure) |
| 83902 | Molecular diagnostics; reverse transcription |

| | |
|---|---|
| 83903 | Molecular diagnostics; mutation scanning, by physical properties (eg, single strand conformational polymorphisms [SSCP], heteroduplex, denaturing gradient gel electrophoresis [DGGE], RNA'ase A), single segment, each |
| 83904 | Molecular diagnostics; mutation identification by sequencing, single segment, each segment |
| 83905 | Molecular diagnostics; mutation identification by allele specific transcription, single segment, each segment |
| 83906 | Molecular diagnostics; mutation identification by allele specific translation, single segment, each segment |
| 83907 | Molecular diagnostics; lysis of cells prior to nucleic acid extraction (eg, stool specimens, paraffin embedded tissue), each specimen |
| 83908 | Molecular diagnostics; amplification, signal, each nucleic acid sequence |
| 83909 | Molecular diagnostics; separation and identification by high resolution technique (eg, capillary electrophoresis), each nucleic acid preparation |
| 83912 | Molecular diagnostics; interpretation and report |
| 83913 | Molecular diagnostics; RNA stabilization |
| 83914 | Mutation identification by enzymatic ligation or primer extension, single segment, each segment (eg, oligonucleotide ligation assay [OLA], single base chain extension [SBCE], or allele-specific primer extension [ASPE]) |
| 83950 | Oncoprotein; HER-2/neu |
| 88271 | Molecular cytogenetics; DNA probe, each (eg, FISH) |
| 88272 | Molecular cytogenetics; chromosomal in situ hybridization, analyze 3-5 cells (eg, for derivatives and markers) |
| 88273 | Molecular cytogenetics; chromosomal in situ hybridization, analyze 10-30 cells (eg, for microdeletions) |
| 88274 | Molecular cytogenetics; interphase in situ hybridization, analyze 25-99 cells |
| 88275 | Molecular cytogenetics; interphase in situ hybridization, analyze 100-300 cells |
| 88291 | Cytogenetics and molecular cytogenetics, interpretation and report |
| 88299 | Unlisted cytogenetic study |
| 88341 | Immunohistochemistry or immunocytochemistry, per specimen; each additional single antibody stain procedure (List separately in addition to code for primary procedure) |
| 88342 | Immunohistochemistry or immunocytochemistry, per specimen; initial single antibody stain procedure |
| 88343 | Immunohistochemistry or immunocytochemistry, each separately identifiable antibody per block, cytologic preparation, or hematologic smear; each additional separately identifiable antibody per slide (List separately in addition to code for primary procedure) |

| | |
|---|---|
| 88344 | Immunohistochemistry or immunocytochemistry, per specimen; each multiplex antibody stain procedure |
| 88360 | Morphometric analysis, tumor immunohistochemistry (eg, Her-2/neu, estrogen receptor/progesterone receptor), quantitative or semiquantitative, per specimen, each single antibody stain procedure; manual |
| 88361 | Morphometric analysis, tumor immunohistochemistry (eg, Her-2/neu, estrogen receptor/progesterone receptor), quantitative or semiquantitative, per specimen, each single antibody stain procedure; using computer-assisted technology |
| 88363 | Examination and selection of retrieved archival (ie, previously diagnosed) tissue(s) for molecular analysis (eg, KRAS mutational analysis) |
| 88364 | In situ hybridization (eg, FISH), per specimen; each additional single probe stain procedure (List separately in addition to code for primary procedure) |
| 88365 | In situ hybridization (eg, FISH), per specimen; initial single probe stain procedure |
| 88366 | In situ hybridization (eg, FISH), per specimen; each multiplex probe stain procedure |
| 88367 | Morphometric analysis, in situ hybridization (quantitative or semi-quantitative), using computer-assisted technology, per specimen; initial single probe stain procedure |
| 88368 | Morphometric analysis, in situ hybridization (quantitative or semi-quantitative), manual, per specimen; initial single probe stain procedure |
| 88369 | Morphometric analysis, in situ hybridization (quantitative or semi-quantitative), manual, per specimen; each additional single probe stain procedure (List separately in addition to code for primary procedure) |
| 88373 | Morphometric analysis, in situ hybridization (quantitative or semi-quantitative), using computer-assisted technology, per specimen; each additional single probe stain procedure (List separately in addition to code for primary procedure) |
| 88374 | Morphometric analysis, in situ hybridization (quantitative or semi-quantitative), using computer-assisted technology, per specimen; each multiplex probe stain procedure |
| 88377 | Morphometric analysis, in situ hybridization (quantitative or semi-quantitative), manual, per specimen; each multiplex probe stain procedure |
| 88384 | Array-based evaluation of multiple molecular probes; 11 through 50 probes |
| 88385 | Array-based evaluation of multiple molecular probes; 51 through 250 probes |
| 88386 | Array-based evaluation of multiple molecular probes; 251 through 500 probes |
| 0007M | Oncology (gastrointestinal neuroendocrine tumors), real-time PCR expression analysis of 51 genes, utilizing whole peripheral blood, algorithm reported as a nomogram of tumor disease index |

| | |
|---|---|
| 0013U | Oncology (solid organ neoplasia), gene rearrangement detection by whole genome next-generation sequencing, DNA, fresh or frozen tissue or cells, report of specific gene rearrangement(s) |
| 0019U | Oncology, RNA, gene expression by whole transcriptome sequencing, formalin-fixed paraffin embedded tissue or fresh frozen tissue, predictive algorithm reported as potential targets for therapeutic agents |
| 0022U | Targeted genomic sequence analysis panel, non-small cell lung neoplasia, DNA and RNA analysis, 23 genes, interrogation for sequence variants and rearrangements, reported as presence/absence of variants and associated therapy(ies) to consider |
| 0036U | Exome (ie, somatic mutations), paired formalin-fixed paraffin-embedded tumor tissue and normal specimen, sequence analyses |
| 0037U | Targeted genomic sequence analysis, solid organ neoplasm, DNA analysis of 324 genes, interrogation for sequence variants, gene copy number amplifications, gene rearrangements, microsatellite instability and tumor mutational burden |
| 0048U | Oncology (solid organ neoplasia), DNA, targeted sequencing of protein-coding exons of 468 cancer-associated genes, including interrogation for somatic mutations and microsatellite instability, matched with normal specimens, utilizing formalin-fixed paraffin-embedded tumor tissue, report of clinically significant mutation(s) |
| 0057U | Oncology (solid organ neoplasia), mRNA, gene expression profiling by massively parallel sequencing for analysis of 51 genes, utilizing formalin-fixed paraffin-embedded tissue, algorithm reported as a normalized percentile rank |
| 0069U | Oncology (colorectal), microRNA, RT-PCR expression profiling of miR-31-3p, formalin-fixed paraffin-embedded tissue, algorithm reported as an expression score |
| 0094U | Genome (eg, unexplained constitutional or heritable disorder or syndrome), rapid sequence analysis |
| 0104U | Hereditary pan cancer (eg, hereditary breast and ovarian cancer, hereditary endometrial cancer, hereditary colorectal cancer), genomic sequence analysis panel utilizing a combination of NGS, Sanger, MLPA, and array CGH, with MRNA analytics to resolve variants of unknown significance when indicated (32 genes [sequencing and deletion/duplication], EPCAM and GREM1 [deletion/duplication only]) |
| 0111U | Oncology (colon cancer), targeted KRAS (codons 12, 13, and 61) and NRAS (codons 12, 13, and 61) gene analysis utilizing formalin - fixed paraffin -embedded tissue |
| G0461 | Immunohistochemistry or immunocytochemistry, per specimen; first separately identifiable antibody |

**Table S3:** Overall survival according to receipt of molecular diagnostic testing, adjusted[c]

| | HR | 95% CI, lower | 95% CI, upper |
|---|---|---|---|
| **Molecular Diagnostic Testing** | | | |
| Yes | 1 | Ref | |
| No | 1.20 | 1.04 | 1.40 |
| **Race** | | | |
| White | 1 | Ref | |
| Black | 1.02 | 0.73 | 1.44 |
| Other | 0.74 | 0.61 | 0.89 |
| **Census Tract Poverty** | | | |
| <10% in poverty | 1 | Ref | |
| >= 10% in poverty | 1.07 | 0.92 | 1.24 |
| **Residence**[a] | | | |
| Metro | 1 | Ref | |
| Urban/Rural | 1.13 | 0.89 | 1.45 |
| **Sex** | | | |
| Male | 1 | Ref | |
| Female | 0.92 | 0.79 | 1.09 |
| **Age at diagnosis (years)** | | | |
| 65-69 | 1 | Ref | |
| 70-74 | 0.92 | 0.73 | 1.15 |
| 75-79 | 0.97 | 0.76 | 1.22 |
| 80-84 | 1.24 | 0.97 | 1.57 |
| 85+ | 1.63 | 1.28 | 2.07 |
| **Charlson comorbidity score** (mean, sd[b]) | 1.13 | 1.07 | 1.19 |
| **Histology** | | | |
| Squamous | 1 | Ref | |

| | | | |
|---|---|---|---|
| Adenocarcinoma | 0.82 | 0.63 | 1.07 |
| Large cell/Other | 1.11 | 0.78 | 1.57 |
| **Stage** | | | |
| III | 1 | Ref | |
| IV | 1.36 | 1.14 | 1.62 |

[a] Residence missing for 7 patients; [b] sd = standard deviation [c] HRs adjusted for all other variables in the table;
